# Supplementary material for: Interacting Environmental Stress Factors Affect Metabolomics Profiles in Stored Naturally Contaminated Maize
Source: Microorganisms. 2022 Apr 20;10(5):853. doi: 10.3390/microorganisms10050853 (PMC9144858; doi:10.3390/microorganisms10050853)
Supplement: Supplementary file 1 [file microorganisms-10-00853-s001.zip › microorganisms-1672053-supplementary.pdf]

Suppl. Table S1. Statistical analyses of the effect of interacting abiotic factors on total secondary metabolites of the three genera found in the maize and the significance of those metabolites produced at > 500 ng/g.

		Wilcoxon/Kruskal-Wallis Tests (Rank Sums)				*Non-parametric Comparisons for each pair using Wilcoxon Method*				
		**Total**	***Aspergillus***	***Fusarium***	***Penicillium***	**Level-Level**	**Total**	***Aspergillus***	***Fusarium***	***Penicillium***
**Total Number of Secondary Metabolites**	Water activity	0.0001*	<.0001*	0.4892	0.0006*	0.95-0.90	0.0245*	<.0001*	0.1541	0.0174*
						0.95-0.85	0.0002*	<.0001*	0.8044	0.0066*
						0.95-0.80	0.0001*	0.0003*	0.6206	0.0174*
						0.90-0.85	0.0005*	0.0021*	0.2688	0.0049*
						0.90-0.80	0.0012*	0.0056*	0.3361	0.0060*
						0.85-0.80	0.6847	0.7447	0.8837	0.9768
	Temperature	0.1527	0.6884	0.0050*	0.0002*	35-30	0.1393	0.2550	0.7100	<.0001*
						35-25	0.0493*	0.5166	0.0252*	<.0001*
						35-20	0.1403	0.6381	0.0593	0.0030*
						30-25	0.3239	0.6834	0.0019*	0.4222
						30-20	0.9754	0.9220	0.0075*	0.7570
						25-20	0.3697	0.4599	0.7488	0.3396
**Total Number of Secondary Metabolites (> 500ng/g)**	Water activity	<.0001*	<.0001*	0.0027*	<.0001*	0.95-0.90	<.0001*	<.0001*	0.0335*	0.0010*
						0.95-0.85	<.0001*	<.0001*	0.0247*	0.0004*
						0.95-0.80	<.0001*	<.0001*	0.0003*	0.0004*
						0.90-0.85	0.0054	0.0707	0.8353	0.0308*
						0.90-0.80	<.0001*	0.5286	0.1605	0.0172*
						0.85-0.80	0.0151*	0.2850	0.0863	0.3861
	Temperature	0.7494	0.4700	0.5504	0.0295*	35-30	0.4961	0.7788	0.3584	0.0057*
						35-25	0.2709	0.5754	0.9766	0.0089*
						35-20	0.8865	0.3450	0.3163	0.1341
						30-25	0.8768	0.2829	0.3920	0.7003
						30-20	0.6647	0.1604	0.7015	0.5518
						25-20	0.6008	06137	0.2753	0.4208

*Indicates significant differences. Different colours indicate the level of significance. Red p-values<0.05, orange p-values<0.001 and black >0.05.
